# Beyond traditional approaches to understanding the functional role of neuromodulators in sensory cortices

**DOI:** 10.3389/fnbeh.2012.00045

**Published:** 2012-07-30

**Authors:** Jean-Marc Edeline

**Affiliations:** Centre de Neurosciences Paris-Sud, CNRS UMR 8195, Université Paris-Sud, BâtimentOrsay Cedex, France

**Keywords:** acetylcholine, cortical processing, electrophysiology, neuromodulators, neuronal selectivity, noradrenaline, norepinephrine, spike-timing

## Abstract

Over the last two decades, a vast literature has described the influence of neuromodulatory systems on the responses of sensory cortex neurons (review in Gu, 2002; Edeline, 2003; Weinberger, 2003; Metherate, 2004, 2011). At the single cell level, facilitation of evoked responses, increases in signal-to-noise ratio, and improved functional properties of sensory cortex neurons have been reported in the visual, auditory, and somatosensory modality. At the map level, massive cortical reorganizations have been described when repeated activation of a neuromodulatory system are associated with a particular sensory stimulus. In reviewing our knowledge concerning the way the noradrenergic and cholinergic system control sensory cortices, I will point out that the differences between the protocols used to reveal these effects most likely reflect different assumptions concerning the role of the neuromodulators. More importantly, a gap still exists between the descriptions of neuromodulatory effects and the concepts that are currently applied to decipher the neural code operating in sensory cortices. Key examples that bring this gap into focus are the concept of cell assemblies and the role played by the spike timing precision (i.e., by the temporal organization of spike trains at the millisecond time-scale) which are now recognized as essential in sensory physiology but are rarely considered in experiments describing the role of neuromodulators in sensory cortices. Thus, I will suggest that several lines of research, particularly in the field of computational neurosciences, should help us to go beyond traditional approaches and, ultimately, to understand how neuromodulators impact on the cortical mechanisms underlying our perceptual abilities.

## Introduction and aims

Despite an abundance of literature, neuromodulators are too often neglected by sensory physiologists. Modern approaches, which aim at dissecting the elementary mechanisms underlying our perceptive abilities, often combine electrophysiological (or optical imaging) results with signal processing and neural network modeling. With this vast range of techniques, we now have rudimentary ideas of mechanisms underlying the perception of complex stimuli such natural scenes or conspecific vocalizations (see review by Huetz et al., 2011 for the auditory modality). However, when trying to dissect the operations conducted by cortical networks, most studies still consider interactions between excitatory (glutamaergic) and inhibitory (GABAergic) input is sufficient to explain how sensory neurons extract the relevant parameters for discriminating between environmental stimuli. For example, in the auditory modality, models aimed at explaining the selectivity of auditory cortex (ACx) neurons for complex sounds and more generally for auditory scene analysis (e.g., Elhilali and Shamma, 2008) systematically involve interactions between excitatory and inhibitory inputs without considering that these selectivities rely, for the most part, on modification by neuromodulators. Implicitly the assumption is that the networks extracting meaningful parameters to discriminate between sensory stimuli would be invariant, leaving no room for the role of the level of attention, arousal, and the learned significance of stimuli in processing this information. Even a short-term plasticity phenomenon such as the “stimulus specific adaptation” Ulanovsky et al. (2003)—viewed as a potential neuronal correlate of habituation or mismatch negativity—is explained by models which only considered interactions between excitatory and inhibitory inputs (Mill et al., 2011). This is in direct contrast with studies aimed at modeling cognitive processes which often envisage a central role for the neuromodulatory systems (Montague et al., 1996; Braver et al., 1999; Yu and Dayan, 2005; Dayan and Yu, 2006).

Before reviewing the existing literature, the first question that should be addressed is: When do neuromodulators influence the processing that takes place in sensory cortices in awake animals? To address this question, electrophysiological recordings performed in behaving animals at the source nuclei of neuromodulatory systems are invaluable tools. Initially, these studies pointed out that neuronal activity in these nuclei depends on the state of vigilance or level of arousal (e.g., see Foote et al., 1980; Aston-Jones and Bloom, 1981). More recent studies clearly indicate that these neurons are also involved in any experiment involving decision processes and/or resolving uncertainty associated with the predictive power of sensory stimuli (Bouret and Sara, 2004; Clayton et al., 2004; review in Bouret and Sara, 2005). These two situations have been explored by experiments describing the action of neuromodulators in sensory cortices. As described in the following sections, a first line of research initially considered that neuromodulators simply provide a level of “arousal”, i.e., a tonic level of excitability by acting on distant extrasynaptic receptors. Increasing the background concentration of neuromodulators during prolonged periods (over tens of seconds or minutes) was, therefore, viewed as mimicking the changes in arousal that occurs when the state of vigilance is modified. In contrast, another line of research evaluated the consequences of phasic activation of neuromodulatory systems using brief stimulation of source nuclei. The immediate effects of phasic activation of neurons, simulates the transient increase in neuromodulator concentrations that should be triggered by brief increases in firing rate, occurring in source nuclei during attentional or learning tasks (Sarter et al., 2009). Taking its roots in these studies, another line of research envisioned that repetition of phasic increases in neuromodulator concentrations alone, is sufficient to promote enduring receptive field and map reorganization. Thus, although a vast amount of literature has described the effects of ACh and NE on the properties of sensory cortex neurons, these studies have been carried out in different frameworks and with different working hypotheses concerning the role of neuromodulators. As described below, this has led to protocols that share apparent similarities but present fundamental differences. In the following sections, examples are taken from the auditory, visual and somatosensory cortices, which share anatomical and functional similarities (see reviews by Edeline, 1999; Guillery and Sherman, 2002). Effects observed in the olfactory system will not be discussed here, but these effects are often, but not systematically, alike those obtained in these three main modalities (review in Linster and Hasselmo, 2001; Giocomo and Hasselmo, 2007).

## Consequences of tonic activation on the selectivity of cortical neurons

As explained above, the increase in neuromodulator concentrations occurring during wide time windows (tens of seconds or of minutes) was presumed to mimic “arousal” or an increase in attention during a state of vigilance. Typically, this strategy has been investigated by continuous iontophoretic application of neuromodulators at the vicinity of the recorded cell. Oddly, this technique revealed that the cholinergic and noradrenergic systems—two major neuromodulatory systems involved in arousal and the waking state—act in opposition to each other. Initial iontophoretic studies reported that application of acetylcholine (ACh) increased the spontaneous firing rate (Krnjevic and Phillis, 1963a,b), whereas application of monoamines such as norepinephrine (NE) decreased it (Krnjevic and Phillis, 1963c). Testing evoked responses in the somatosensory, visual, and auditory cortices confirmed this dichotomy. Application of ACh increased evoked responses (Sillito and Kemp, 1983; Sato et al., 1987; Lamour et al., 1988; Metherate et al., 1988; McKenna et al., 1989), whereas application of NE depressed them (Foote et al., 1975; Videen et al., 1984; Kolta et al., 1987; Manunta and Edeline, 1997). A few studies replicated these findings in the awake animal (Foote et al., 1975; Bassant et al., 1990a,b; Manunta and Edeline, 1999), which argues against the possibility that anesthesia changes the balance between depolarization and hyperpolarization induced by ACh and NE. Moreover, it was shown in awake rats that continuous low frequency stimulation (1 Hz) of Locus Coeruleus (LC) neurons trigger similar effects to those seen with continuous iontophoretic application: tonic activation of LC neurons decreased evoked responses in 63% of the cells in the rat somatosensory cortex (Devilbiss and Waterhouse, 2004). It has been argued that the inhibitory effects induced by NE iontophoretic application were a consequence of too high concentrations of NE at the vicinity of the cell (Waterhouse et al., 1998a,b). This seems unlikely given that (1) pronounced depression of evoked responses were also observed with very low ejection currents (Manunta and Edeline, 1997; Ego-Stengel et al., 2002); (2) decreased responses were also obtained with stimulation of the LC (Lecas, 2004; Edeline et al., 2011) and (3) no biphasic effect has been reported when *in vitro* studies have tested synaptic inputs converging on a given cell^1^ (Law-Tho et al., 1993; Pralong and Magistretti, 1994, 1995). An alternative explanation (that remains to be tested) is that iontophoretic ejection can affect, or not, local inhibitory interneurons projecting onto the recorded cells, leading to decreases or increases depending upon the proportions of direct vs. indirect effects on the recorded cell. Support for this has come from recent studies conducted in the ACx showing NE can affect inhibitory interneurons (Salgado et al., 2011, 2012).

### What are the functional consequences on cortical neurons?

**Figure 1:**
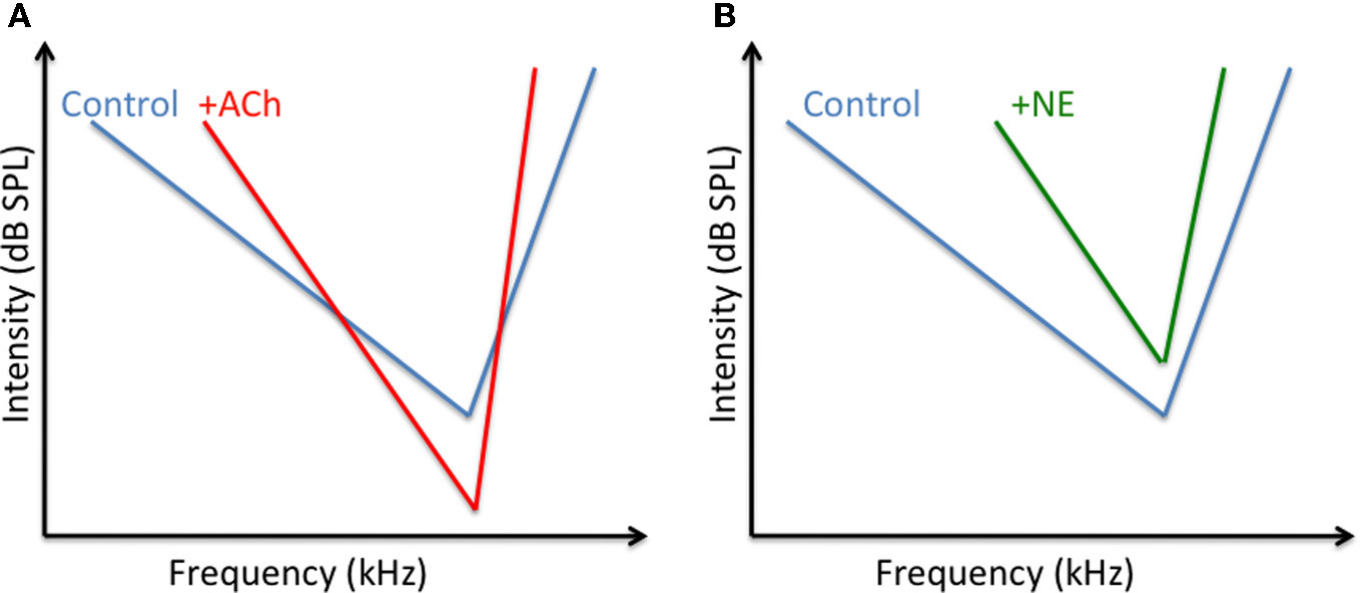
**Potential changes of ACh and NE on the tuning curve of auditory cortex neurons. (A)** Converging results (Ashe et al., 1989; McKenna et al., 1989; Metherate et al., 1990; review in Metherate, 2011) indicate that ACh decrease the acoustic threshold at the CF and at surrounding frequencies whereas it should increase the threshold for frequency far away from the CF. These differential effects should lead to a sharpening of the tuning curve. **(B)** Converging results (Manunta and Edeline, 1997, 1998, 1999, 2000); suggest that NE increases the acoustic threshold at the CF and at surrounding frequencies. The increases in threshold is lower at the CF than at frequencies far away from CF, which also leads to a sharpening of the tuning curve.

Contrasting with their opposite effects on response strength, ACh and NE both improve the neuronal selectivity for a particular dimension of the stimulus. In the visual cortex, application of ACh enhanced the orientation and direction selectivity (Sillito and Kemp, 1983; Murphy and Sillito, 1991; but see Zinke et al., 2006 for opposite effects)^2^. Recent studies have tried to clarify the effects of ACh on the relationship (gain) between the stimulus contrast (input) and the response magnitude (output). Iontophoretic application of ACh in primate visual cortex modulated the response gain control, but not the contrast gain control, meaning that the greatest contrast evoked the strongest responses whereas the contrast evoking half the maximal response, remained unaffected (Disney et al., 2007; Soma et al., 2012). This effect of the contrast response function was prominent in layer 4 and was sometimes accompanied by a decrease in threshold contrast (Disney et al., 2007). In some studies, the facilitatory effect of ACh appears to be mediated only by nicotinic receptors (Disney et al., 2007) whereas in other studies the facilitatory effect were mediated by muscarinic (mChRs) and by nicotinic (nChR) receptors (Soma et al., 2012). In ACx, application of ACh (or anticholinesterase) facilitated the response for the neurons' best frequency and for the adjacent frequencies, without generating general changes across frequency tuning (Ashe et al., 1989; McKenna et al., 1989). These effects were also accompanied by a lower acoustic threshold in the presence of ACh (Metherate et al., 1990). Figure 1A summarizes the cholinergic effects in the ACx.

Iontophoretic application of NE also revealed that this neuromodulator can improve the functional properties of neurons in several cortical areas. In ACx, the suppressive effect of NE promoted an increase in frequency selectivity in anesthetized and unanesthetized animals (Edeline, 1995; Manunta and Edeline, 1997, 1999) and an increase in acoustic threshold (Manunta and Edeline, 1998). Figure 1B summarizes these effects. In the visual cortex, application of NE improved the velocity and direction selectivity of cells, without modifying the orientation selectivity (McLean and Waterhouse, 1994). This lack of effect on orientation selectivity was confirmed despite strong attenuation of evoked responses (Ego-Stengel et al., 2002). To account for these results, it was proposed that, contrary to a subtractive effect, which would lead to an increase in selectivity, the action of NE is rather a divisive effect (i.e., a gain control), which affects the level of cortical responsiveness without affecting the functional selectivity. These results point out that the effects of NE (and maybe of any other neuromodulator) could potentially differ depending on the stimulus dimension. For example, a dimension that depends on thalamo-cortical afferences (such as frequency tuning in the ACx, or the size of the receptive field in the visual cortex) could be more affected than a dimension that relies more on the cortico-cortical afferences (such as frequency modulation tuning in the ACx, or the velocity tuning in the visual cortex). This possibility is supported by the fact that testing different inputs converging on the same cortical location reveal that both NE and ACh can strongly attenuate synaptic responses of one input while exerting less suppression, or even enhancing, responses of the other input (Hasselmo et al., 1997; Hsieh et al., 2000).

### What are the cellular mechanisms underlying these receptive field modifications?

**Figure 2:**
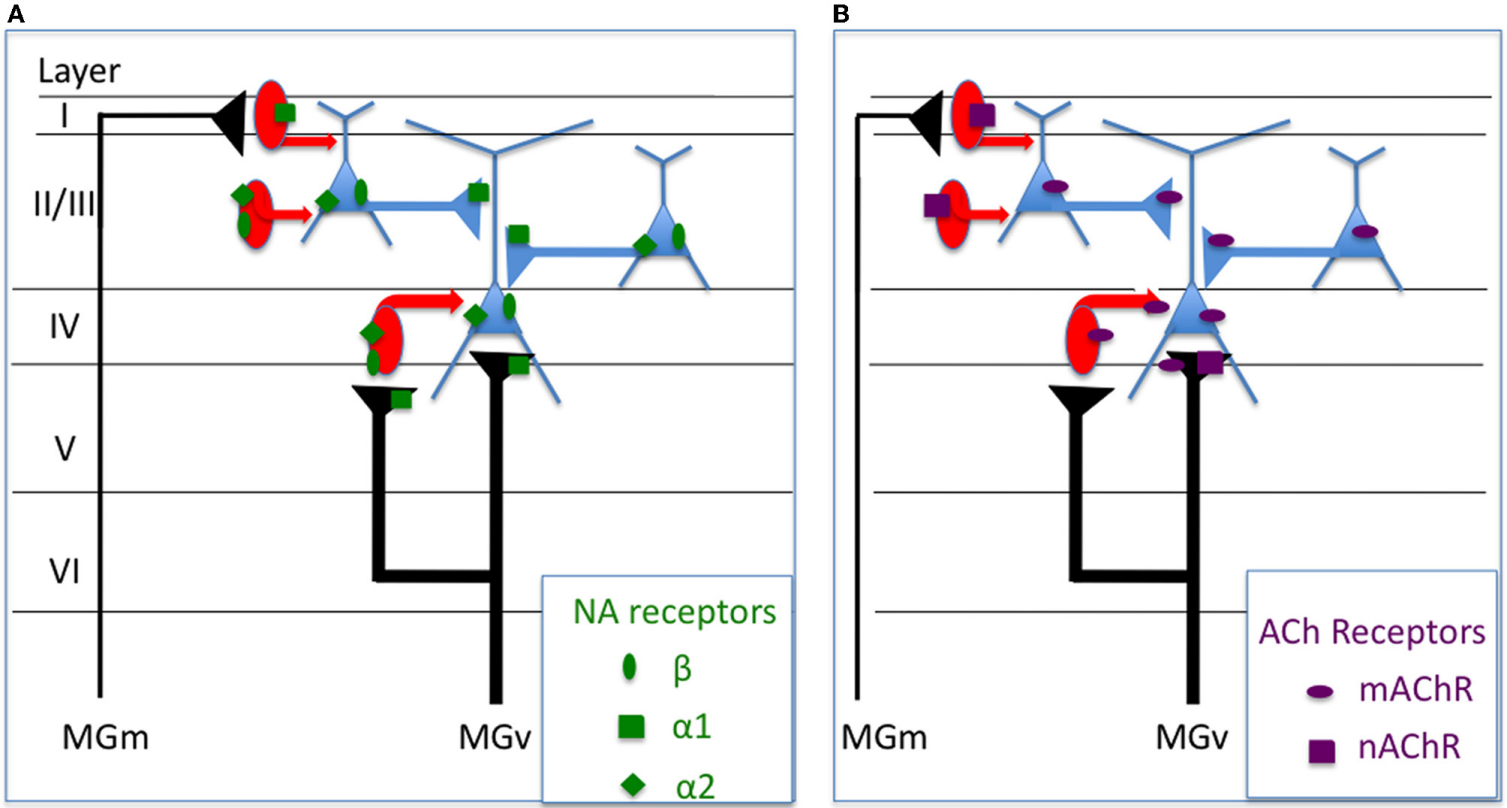
**Potential mechanisms underlying the physiological effects of NE and ACh on sensory cortex neurons**. The summary presented here is mostly based on findings obtained in the visual and auditory cortex. **(A)** Alpha1 noradrenergic receptors can control the glutamaergic transmission (thalamo-cortical and cortico-cortical) by effects occurring at the presynaptic level. They can also control the excitability of layer I inhibitory interneurons (Salgado et al., 2011). Beta and alpha2 receptors can both contribute to control the post-synaptic excitability of cortical cells (Manunta and Edeline, 1997, 1999; Salgado et al., 2011). **(B)** Muscarinic receptors increase the post-synaptic excitability of pyramidal cells (Metherate et al., 1992; Cox et al., 1994) but also of some types of interneurons (Disney and Aoki, 2008) and can decrease the release of GABA by Fast-Spiking interneurons (Kruglikov and Rudy, 2008). Nicotinic receptors can act presynaptically on the thalamo-cortical and can increase the excitability of several types of GABAergic interneurons (Disney et al., 2007; review in Metherate, 2004, 2011).

*In vivo* studies have shown that activation of mChR receptors enhance cortical responses to sensory inputs (Metherate et al., 1988; McKenna et al., 1989; Chen and Yan, 2007). In general, this activation increases post-synaptic membrane resistance (due to decreased potassium conductances) and leads to an increase in post-synaptic excitability via a slow EPSP and a decrease in the after hyperpolarization potential (Metherate et al., 1992; Cox et al., 1994). However, parts of these effects can also be evoked by muscarinic receptors located on particular types of interneurons (Disney and Aoki, 2008): it has been shown that activation of muscarinic receptors can increase neuronal responses by decreasing the release of GABA from interneurons in layers II/III (Salgado et al., 2007) and layer V (Kruglikov and Rudy, 2008). Also, several recent studies suggest that nicotinic receptors (nChR) also contribute to receptive field modulation (see above) which can be mediated by both presynaptic regulation of thalamocortical transmission and by a postsynaptic increase in excitability of several types of GABAergic interneurons (Disney et al., 2007; Letzkus et al., 2011 review in Metherate, 2004, 2011). Taken together, these data indicate that both nicotinic and muscarinic mechanisms sculpt the neurons' receptive fields by affecting differentially the inhibitory interneurons vs. the pyramidal cells (see Figure 2B).

The mechanisms underlying the noradrenergic effects are particularly difficult to dissect. In the somatosensory cortex, some of the observed facilitation induced by NE has been shown to be replicated by application of alpha1 agonists (Devilbiss and Waterhouse, 2000; Waterhouse et al., 2000) whereas some NE-induced decreased responses were replicated by Beta agonists. In contrast, in the ACx, decreased responses produced by application of NE were systematically blocked by alpha1 antagonists, and the increased responses by beta antagonists (Manunta and Edeline, 1997, 2004; Dinh et al., 2009). In fact, a single ACx neuron can display both a decrease in the evoked response during activation of presynaptic alpha1 receptors and a facilitation of the evoked response by activation of beta receptors (Manunta and Edeline, 1997, 2004). Thus, in the ACx, the balance between alpha1 and beta receptors present at the pre- and post-synapse might determine the net effect induced by NE on a given neuron (see Figure 2A). Nonetheless, studies in other cortical areas suggested that other receptors potentially underlie the decrease in evoked responses induced by NE. For example, in the visual cortex, NE-induced inhibition can be replicated by alpha2 agonists (Kolta et al., 1987) and, similarly, in the entorhinal cortex, the NE-induced decrease of fast and slow excitatory postsynaptic currents (EPSP) can be blocked by alpha2 antagonists (Pralong and Magistretti, 1994, 1995). In the ACx, NE decreases the EPSC amplitude for 25–50 min, an effect that is mimicked by alpha1 agonists (Dinh et al., 2009). In the same cortex, NE acts differentially on the inhibitory effect occurring in different cortical layers: alpha1 receptors mediate a decrease in IPSCs evoked by stimulation of layer I whereas beta and alpha2 receptors increase the amplitude of IPSCs evoked by stimulation of layer II/III (Salgado et al., 2011). This echoes the study by Kawaguchi and Shindou (1998) who reported that NE can depolarize some, but not all, interneuron types with or without affecting the neurons firing rate. To the best of our knowledge, there has been no demonstration *in vivo* that shows NE acts principally on inhibitory interneurons, and the depressive effects NE has on evoked responses could be observed in the presence of bicuculline (see Figure 10 in Manunta and Edeline, 1997 and Figure 7 in Manunta and Edeline, 1998). This suggests that the depressive effects of NE in sensory cortices are not systematically mediated by GABAergic neurons, as indicated by studies showing that EPSC's are reduced by NE application (Law-Tho et al., 1993; Dinh et al., 2009).

## Phasic activation of the cholinergic and noradrenergic systems

The second strategy to uncover the role of neuromodulators in sensory cortices is to consider that phasic activation of source nuclei mimic their influence when neuromodulatory systems react to environmental stimuli. A vast number of studies have described the facilitatory effects produced by associating a sensory stimulus with activation of the nucleus basalis magnocellularis (NBM), the unique source of cortical ACh. Both in the somatosensory (Rasmusson and Dykes, 1988; Tremblay et al., 1990; Webster et al., 1991) and the ACx (Metherate and Ashe, 1991; Hars et al., 1993; Edeline et al., 1994a,b; Bakin and Weinberger, 1996; Bjordahl et al., 1998; Dimyan and Weinberger, 1999) such pairing protocols facilitate sensory responses. This effect has been demonstrated using both evoked potentials (Rasmusson and Dykes, 1988; Metherate and Ashe, 1991) and unit recordings (Tremblay et al., 1990; Hars et al., 1993; Edeline et al., 1994a,b).

Fewer studies have been conducted using stimulation of the LC. Initial studies reported that LC stimulation predominantly decreased neuronal activity in several cortical areas including in visual cortex (Olpe et al., 1980; Sato et al., 1989). In the somatosensory cortex, both the excitatory and the inhibitory components of evoked responses were facilitated when phasic stimulation of the LC was delivered 200–300 ms before tactile stimuli (Waterhouse et al., 1998a,b; Snow et al., 1999). In fact, in this cortex, LC stimulation strongly impacts on the LC stimulation shortened evoked responses and reduced the first spike latency and its variance (Lecas, 2001, 2004). An impact of LC stimulation on both the strength and temporal organization of the response has also been observed in the olfactory cortex (Bouret and Sara, 2002). In ACx, facilitation of evoked responses was the dominant effect when stimulation of the LC was delivered 250 ms before a pure tone (Edeline et al., 2011). Comparing these results with those in which phasic pulses (1 s) of NE were delivered in the vicinity of the recorded cells (Manunta and Edeline, 2004) indicates that the effects produce by LC stimulation and NE application differ: the percentage of facilitated responses was twice as high with LC stimulation than with iontophoretic application (53% vs. 21%). Importantly, the same applies to ACh: when phasic pulses of ACh were paired with a pure tone, decreased evoked responses were prevalent (Metherate and Weinberger, 1989, 1990) whereas increases were systematically observed with NBM stimulation (Bakin and Weinberger, 1996; Bjordahl et al., 1998; Dimyan and Weinberger, 1999). Thus, despite their opposite effects with iontophoretic application, activation of the whole cholinergic or noradrenergic systems facilitates sensory evoked responses of cortical neurons with the same potential to produce facilitation that is selective for the stimulus associated with LC/NBM activation. Several classical reasons are proposed for explaining the differences between iontophoretic studies and stimulation of source nuclei: (1) the NBM area contains non-cholinergic neurons which could be involved in inducing the observed effects; (2) the LC neurons express a variety of neuropeptides (vasopressin, somatostatine, neuropeptide Y, enkephalin, neurotensin, and galanin) which can generate a diverse range of effects on cortical cells and modify the effects of NE *per se*. Last, we should keep in mind that at the level of the recorded neuron, the release of neuromodulators radically differs with these two techniques: after NBM/LC stimulation, ACh/NE is diffusely released from hundreds of synapses or varicosities which impact on the entire dendritic tree and is fundamentally different from the release from a iontophoretic pipette arbitrary placed at a given distance from the soma. Also, it is possible that the higher excitability observed after NBM or LC stimulation results from activation of high affinity receptors on cell bodies with low concentrations of NE/ACh, while more complex synaptic effects take place at higher concentrations of NE/ACh, as seen with iontophoretic applications. Table 1 summarizes the main results obtained with NE and ACh.

**Table 1:** **Summary of the main *in vivo* experiments testing the effects of the noradrenergic or cholinergic modulation in sensory cortices**.

	**Cortical area/species**	**System or drug**	**Technique**	**Effects on evoked responses**	**Dominant effect on functional properties of cortical cells**
Foote et al., 1975	AI/monkey	NE	Ionto	↓	Not quantified
Sillito and Kemp, 1983; Murphy and Sillito, 1991	V1/cat	ACh	Ionto	↑	Not quantified
Videen et al., 1984	V1/cat	NE	Ionto	↓	Not quantified
Kolta et al., 1987	V1/rat	NE	Ionto	↓	Not quantified
Sato et al., 1987	V1/cat	ACh	Ionto	↑	No change in orientation/direction selectivity
Lamour et al., 1988	S1/rat	ACh	Ionto	↑	Increase in receptive field size
Metherate et al., 1988	S1/cat	ACh	Ionto	↑	Not quantified
Metherate et al., 1990	AI/guinea pig		Ionto	↑	Decrease in acoustic threshold
Ashe et al., 1989; McKenna et al., 1989	AI/cat	ACh	Ionto	↑	Frequency tuning changes by differential effects on the BF vs. non-BF responses
Sato et al., 1989	V1/rat	NE	LC stim	↓	Not quantified
Bassant et al., 1990a,b			Ionto		Not quantified
Rasmusson and Dykes, 1988; Tremblay et al., 1990; Webster et al., 1991	S1/cat, rat, racoon	ACh	NB stim	↑	Not quantified
Metherate and Weinberger, 1989, 1990	AI/cat	ACh	Ionto	↓	Frequency specific decrease in the frequency tuning after pairing
Hars et al., 1993; Edeline et al., 1994a,b	AI/rat	ACh	NB stim	↑	Not quantified
McLean and Waterhouse, 1994	V1/rat	NE	Ionto	↓	Sharpening of the velocity and direction tuning with no effects on the orientation tuning
Waterhouse et al., 1998a,b	S1/rat	NE	LC stim	↑	Not quantified
Bakin and Weinberger, 1996; Bjordahl et al., 1998; Dimyan and Weinberger, 1999	AI/guinea pig	ACh	NB stim	↑	Selective facilitation in the frequency tuning for the frequency paired with the NB stimulation
Manunta and Edeline, 1997, 1998, 1999, 2000	AI/rat AI/guinea pig	NE	Ionto	↓	Sharpening of the frequency tuning and increase in threshold
Waterhouse et al., 1998a,b	S1/rat	NE	LC stim	↑	Not quantified
Lecas, 2001, 2004	S1/rat	NE	LC stim	↓	Not quantified
Ego-Stengel et al., 2002	V1/cat	NE	Ionto	↓	Sharpening of the orientation and direction selectivity
Manunta and Edeline, 2004	AI/rat		Ionto	↓	Selective ↓ at the paired frequency
Devilbiss and Waterhouse, 2004	S1/rat	NE	LC stim	↓	Not quantified
Devilbiss et al., 2006; Devilbiss and Waterhouse, 2011	S1/rat	NE	LC stim	↑	Increase between cells cross-correlations
Zinke et al., 2006	V1/monkey	ACh	Ionto		Broadening of orientation tuning
Disney et al., 2007	V1/monkey	ACh	Ionto	↑	Lower detection threshold for visual stimuli
Edeline et al., 2011	AI/rat	NE	LC stim	↓ and ↑	Selective effects at the paired frequency
Soma et al., 2012	V1/monkey	ACh	Ionto	↑	Increase the response gain of the contrast-response function

## Phasic activation as a way to trigger receptive field and map reorganization

A natural extension of the experiments mentioned above is to consider that any learning situation involves a repeated and systematic association between a particular stimulus and the activation of neuromodulatory systems. This line of research generally aims at investigating whether a particular neuromodulatory system plays a major role in learning-induced long-term sensory plasticity (reviewed in Edeline, 2003). In this field, the ACx is probably the cortical area where the effects of ACh have been the most extensively documented (review in Weinberger, 2004, 2007) and there is now compelling evidence that the cholinergic system can promote receptive field and map reorganizations. At the single cell level, repeated associations between a particular sound frequency and phasic activation of the NBM retune ACx neurons to that frequency both in anesthetized and awake animals (Bakin and Weinberger, 1996; Bjordahl et al., 1998); an effect that was not observed when the association was made, using muscarinic antagonists (Bakin and Weinberger, 1996; Miasnikov et al., 2008). When such a pairing regimen was continued over 20–25 days (with each day hundreds of associations between NBM stimulation and a particular sound frequency), the map of primary ACx exhibits a massive over-representation favoring that frequency (Kilgard and Merzenich, 1998a). In addition, when the stimulus associated with NBM stimulation was a particular rate of presentation (5, 7.5, or 15 Hz), ACx neurons favors that particular rate (Kilgard and Merzenich, 1998b). Similarly, when a particular temporal frequency of whisker deflection was associated with ACh application, long-lasting and selective facilitation was observed for that particular frequency (Shulz et al., 2000). These two results suggest that ACh can alter the temporal selectivity (indexed in terms of firing rate) of cortical networks.

Not surprisingly, activation of other neuromodulators can also produce cortical map reorganizations similar to those triggered by the cholinergic system: activating the dopaminergic system (by stimulating the ventral tegmental area) also generates massive cortical reorganizations in primary and non-primary ACx (Bao et al., 2001, 2003). Little is know about the cellular mechanisms underlying this cortical remodeling: the effects induced by the dopaminergic system can be antagonized by D1 or D2 receptor antagonists but as yet there have been no pharmacological controls conducted with stimulation of the NBM (Kilgard and Merzenich, 1998a,b) despite the fact that this area is known to contain numerous non-cholinergic neurons (Jones and Muhlethaler, 1999; Zaborszky et al., 1999; review in Sarter and Bruno, 2002) which can potentially contribute to map reorganization (see for discussion on this point Ramanathan et al., 2009).

Ideally, the mechanisms underlying plasticity induced by neuromodulators should be studied *in vivo*. However, to date, most, if not all the data on these mechanisms are from *in vitro* studies. Initial studies have reported that NE favors the probability of LTP induction in the visual cortex (e.g., Bröcher et al., 1992) and promote the occurrence of LTD in conditions of paired-pulse stimulation that do not normally promote LTD (Kirkwood et al., 1999). More recently, several groups have investigated how neuromodulators influence spike-timing dependent plasticity (STDP). For example, it has been shown that associative pairing protocols given during beta-adrenergic receptors activation systematically lead to LTP, independently of the timing relationship between the test and the conditioning pathway (Seol et al., 2007). In fact, it seems that all receptors coupled to adenyl cyclase pathway enable associative LTP, whereas receptors coupled to the phospholipase C cascade enable the induction of LTD regardless of the order of pre- and post-synaptic activation (Seol et al., 2007). Neuromodulators can influence STDP rules in several ways. They can change the shape of the STDP temporal window, allowing for longer pre-post timing delays that increases synaptic efficacy (Zhang et al., 2009). They can also change the conditions for plasticity by either increasing (Couey et al., 2007) or decreasing the threshold for induction of plasticity (Lin et al., 2003; Zhang et al., 2009). More surprising, specific manipulations of one or several neuromodulators can result in sign reversal of plasticity, i.e., stimulation patterns that would normally induce LTP can promote LTD and vice-versa (Bissiere et al., 2003; Couey et al., 2007; Seol et al., 2007; Zhang et al., 2009). Lastly, some neuromodulators can exert short- or long-term, effects on dendritic excitability and backpropagation of action potentials which will affect the efficacy of the STDP protocol (see review by Pawlak et al., 2010).

To conclude this section, it seems that phasic activation of the source nuclei of neuromodulators is now considered as a powerful tool to trigger map reorganization. This demonstrates that activating some neuromodulatory systems is sufficient to produce large-scale synaptic plasticity that underlies map reorganization. This does not mean that all types of map plasticity are controlled by neuromodulators: The cholinergic system has long been viewed as controlling cortical reorganization as excitotoxic (Juliano et al., 1991) or immunotoxic (Conner et al., 2003, 2005) lesions of NMB area prevent experience-dependant map reorganizations. However, recent studies point out that NBM immunotoxic lesions do not prevent ACx reorganization after cochlear lesions (Kamke et al., 2005; see also Ramanathan et al., 2009).

## The diversity of neural codes operating in sensory cortices

It is now commonly accepted that the neural code underlying the perception of sensory stimuli does not solely rely on the number of action potentials elicited by presentation of sensory stimuli. Over the last 20 years, a growing literature has described the contribution of the temporal organization of spike trains to the neural code operating in sensory cortices (review in Theunissen and Miller, 1995; Bair and Koch, 1996; Rieke et al., 1997; Buracas et al., 1998; Mechler et al., 1998; Borst and Theunissen, 1999; Huetz et al., 2011). The strict definition of what should be considered as a temporal code *sensus stricto* has been and continues to be the subject of endless debate: Should we talk about the temporal code whenever analysis reveals that the responses temporal organization contains more information about the identity of the stimulus than does the firing rate, or should we talk about the temporal code only when there is no temporal information contained in the stimulus (see for discussion Borst and Theunissen, 1999)? Initially, the most convincing evidence in favor of the later point came from studies conducted in area MT by Richmond and colleagues using static stimuli (Optican and Richmond, 1987; Richmond et al., 1987a; Richmond and Optican, 1987b). Similarly, in the ACx, a set of studies conducted by Middlebrooks and colleagues demonstrated that the temporal organization of neuronal discharges can provide a panoramic code for sound localization (Middlebrooks et al., 1994, 1998; Furukawa and Middlebrooks, 2002; Mickey and Middlebrooks, 2003). Subsequent studies have shown that temporal sequences of action potentials (involving triplets or quadruplets) occur above chance level and contained information about the stimulus (Oram et al., 1999; Richmond et al., 1999; see also Wiener and Richmond, 2003 for discussion). To assess the precision of temporal coding operating in visual cortex, Victor and Purpura (1996, 1997) have designed a new method which allows for estimation of information contained in spike trains and avoids the classical binning problem of the “direct” method (Strong et al., 1998) when estimating this information (discussed in Victor, 2000). It appeared that most (83%) of the neurons of V1 and V2 contained a significant amount of information when temporal organization is considered, whereas only 48% of the cells contained information about the stimulus orientation or the contrast when only considering the number of action potentials. Applying Victor and Purpura's method to spike trains in ACx neurons recorded during presentation of conspecific vocalizations indicate that few cortical cells (10%) displayed firing rate preference for vocalization, whereas many cells (75%) displayed a significant amount of information about vocalizations when the information transmitted by spike trains was quantified with a temporal precision of 10–50 ms, particularly in awake conditions (Huetz et al., 2009). Similar results have been shown with slightly different methods in different species. In the ACx of ferrets (Schnupp et al., 2006), or its analogous structure in birds (Narayan et al., 2006), information on the stimulus identity obtained with a precision of about 10 ms is much higher than that based on the overall firing rate. In addition, the neurometric curves obtained with temporal spike patterns, exhibited a relatively good match with the psychometric curves. However, the neurometric curves obtained with spike rate did not (Narayan et al., 2007; Walker et al., 2008), which suggests that neural mechanisms operating on time scale of 10 ms might underlie behavioral discrimination. This is not specific to the auditory modality, as data from the visual cortex indicates that primary visual cortex neurons also display a surprisingly low degree of spike time variability when stimulated with natural images (e.g., see Yen et al., 2007; Haider et al., 2010; Herikstad et al., 2011; reviewed in Kayser et al., 2004).

Needless to say, the neural code underlying the perception of sensory stimuli involves interactions between vast populations of neurons distributed throughout cortical and subcortical networks. Based on decades of research from leading groups, neuronal interactions during presentation of sensory stimuli are now commonly described. For example, in the auditory system, correlations can emerge with acoustic stimulation whereas they do not exist in spontaneous activity (Dickson and Gerstein, 1974; deCharms and Merzenich, 1996). Neuronal interactions are also stimulus-dependent (Eggermont et al., 1983; Frostig et al., 1983; Espinosa and Gerstein, 1988; deCharms and Merzenich, 1996; Gourévitch and Eggermont, 2007) and can code for stimulus parameters where the firing rate is insensitive (e.g., movement of a sound, see Ahissar et al., 1992).

Over the last decade, the development of silicon probes and spike sorting methods have allowed for simultaneous recording of the activity of tens of neurons *in vivo*, and, therefore the ability to detect the synchronization of cell assemblies and their modulation by the presentation of sensory stimuli (Buzsaki, 2004). Large-scale neuronal recordings have shown that spatiotemporal patterns, with a precise temporal organization, do occur with the presentation of sensory stimuli. In the ACx, these patterns differ between the classes of stimuli (e.g., pure tones vs. natural sounds) and are also detected during spontaneous activity (Luczak et al., 2009; Sakata and Harris, 2009). Similar findings have been reported in the visual cortex (Jermakowicz et al., 2009). That these patterns do exist during spontaneous activity suggests that the temporal dynamics of spiking patterns is determined by anatomical constraints involving both local circuit properties and more widespread circuitry (intra areal or inter-areal) interactions (see for discussion Harris et al., 2011).

## Do neuromodulators affect the temporal aspects of the neural code?

**Figure 3:**
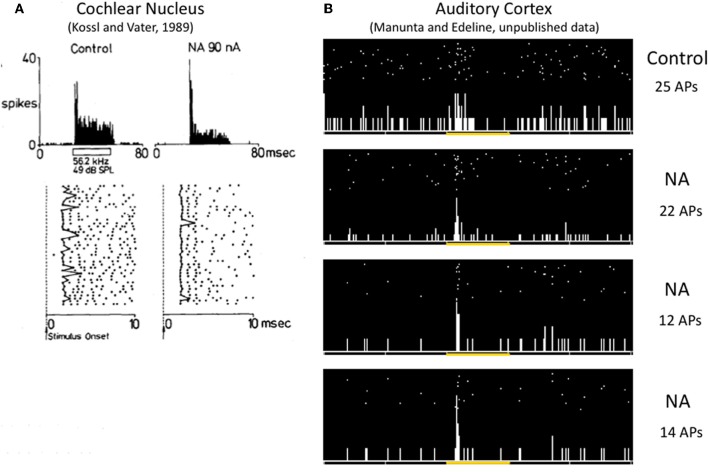
**Effects of NE on the variability of first spike latency in the cochlear nucleus and the auditory cortex. (A)** In the anteroventral cochlear nucleus, iontophoretic application of NE at the vicinity of this cell reinforced the phasic component of the response by reducing the jitter of the first spike latency (as it can be observed on the raster, data from Kössl and Vater, 1989). **(B)** In the auditory cortex, iontophoretic application of NE (1 s pulses of NE repeated 30 times) at the vicinity of this cell transformed a weak disorganized response into a brief phasic response, mostly by reducing the jitter of the first spike latency (unpublished results from Manunta and Edeline).

**Figure 4:**
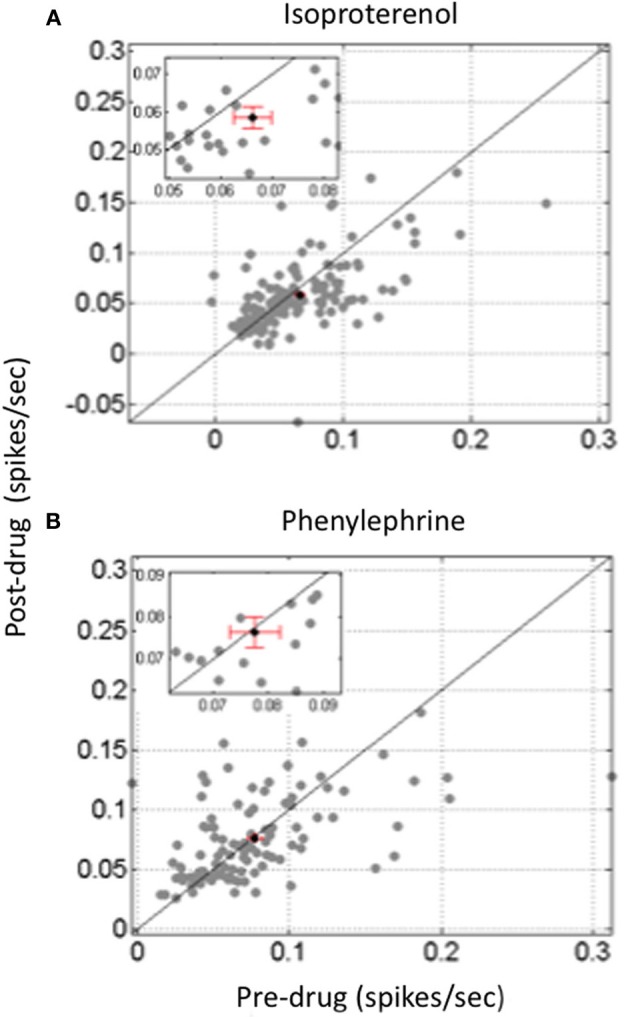
**Differential effect of beta and alpha1 agonist on between cells cross-correlations in the auditory cortex**. Scattergrams representing the value of the mean cross-correlation obtained pre-drug application against the value obtained after drug application. Data are based on simultaneous recordings collected in less than 400 μm apart in layer III or IV of the guinea pig auditory cortex. **(A)** Isoproterenol, a classical Beta agonist, increased evoked activity but significantly decreased the value of between cell cross-correlations. **(B)** In contrast, Phenylephrine, a classical Alpha1 agonist, decreased evoked activity but had not significant effect on the value of between cell cross-correlations.

So far, only a few studies have investigated to what extent the different facets of the neural code operating in the thalamocortical sensory systems are affected by neuromodulators. The paucity of the results is such that it is quite difficult to draw general conclusions. A few studies have pointed out that NE modulates the timing of neuronal responses. For example, in the anteroventral cochlear nucleus, iontophoretic application of NE decreased the latency variability of evoked responses (Kössl and Vater, 1989), an effect that can also be observed at the cortical level (Manunta and Edeline unpublished observations, see Figure 3) Furthermore, in somatosensory cortex, simultaneous analyses of current source density and single unit responses showed that phasic activation of LC neurons produced (i) a compression of the supragranular sink responses (which appeared sooner and had a shorter duration than in control situation) and (ii) a reduction of both single unit response latency and its variability (Lecas, 2001, 2004). Recently, the consequences of phasic or tonic LC activation on the synchronization of an ensemble of recordings in the somatosensory thalamocortical system have been described. Phasic activation of the LC produced a two-fold increase in the number of significant between cell cross-correlations and this was accompanied by a four-fold increase in the mean correlation strength. Surprisingly, this effect was prominent at the thalamic level but was rarely observed at the cortical level (Devilbiss and Waterhouse, 2011). The functional connectivity between pairs of thalamic and cortical neurons was modestly increased both in terms of the number of significant correlations and in terms of the mean correlation strength. Weaker effects were obtained with tonic activation of the LC (Devilbiss et al., 2006; Devilbiss and Waterhouse, 2011). Interpreting an increase in cross-correlation between thalamic cells is not trivial given the lack of direct anatomical connectivity between somatosensory thalamus neurons (Barbaresi et al., 1986) except di-synaptically, via the thalamic reticular nucleus (Crabtree et al., 1998). In fact, the most parsimonious explanation is that LC activation enhances synchronization of common excitatory inputs converging onto thalamic cells. These common inputs can stem from the trigeminal nucleus, the somatosensory cortex, or both. Recently, we have observed that different noradrenergic receptors have distinct effects on cross-correlations between ACx neurons (Figure 4). Activation of Beta receptors (which increases evoked responses) decreases the between cell cross-correlations whereas activation of alpha1 receptors (which decreases evoked responses) does not change cross-correlations (Gaucher et al., 2012)^3^ These effects occurred for cells recorded at short distances (<400 μm) not over the entire ACx. Similarly, iontophoretic application of ACh rarely produced significant changes in cross-correlation between cortical neurons, but when it did, there were no concomitant changes in average firing rate, suggesting that cholinergic modulation can affect cortico-cortical connections without affecting neuronal excitability (Shulz et al., 1997). Thus, it seems that both with NE and ACh, changes in firing rate cannot predict modulation of between cell interactions.

## Conclusions and perspectives

**Figure 5:**
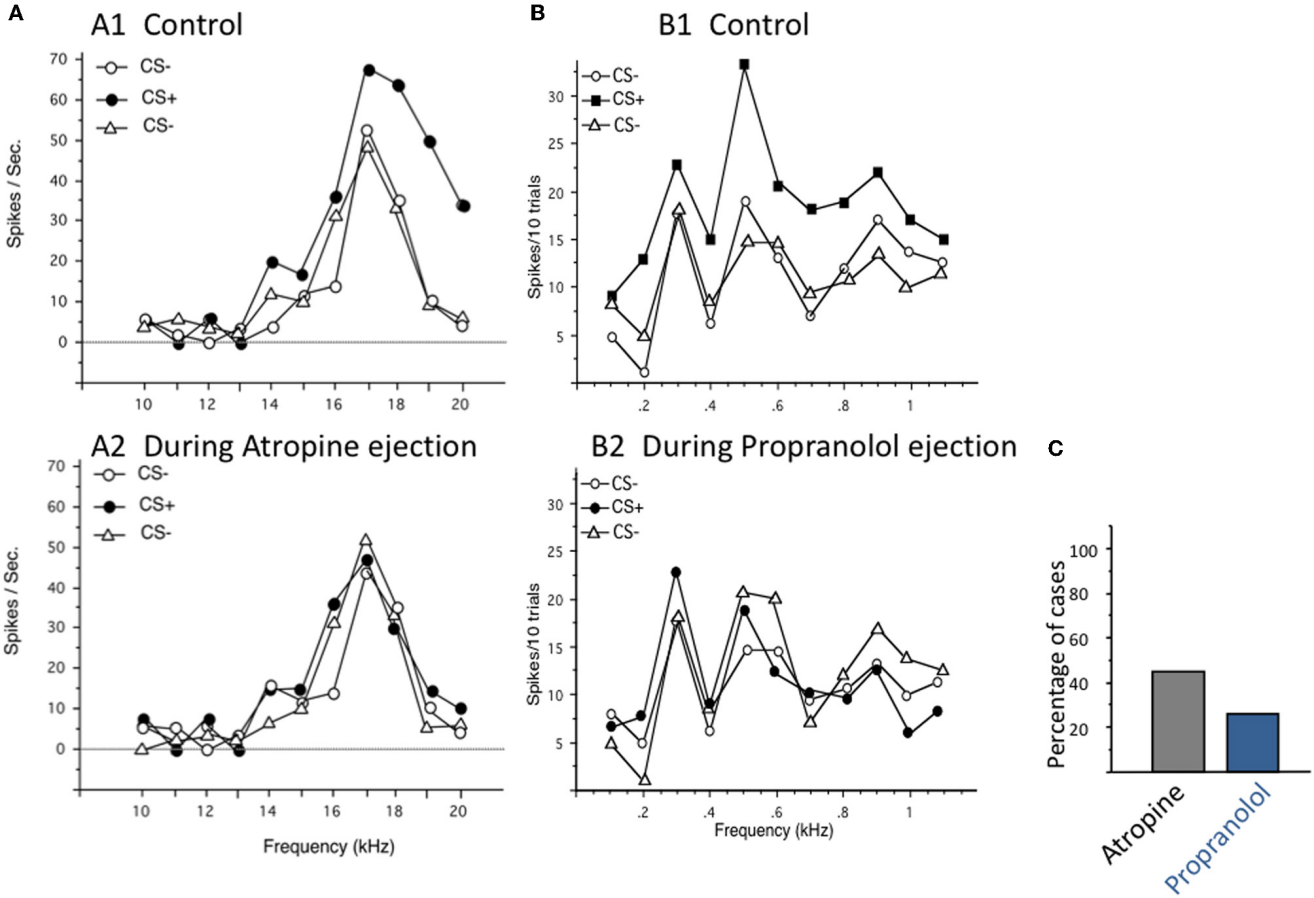
**Task-related changes in firing rate can be blocked either by cholinergic or by noradrenergic antagonists**. In that protocol, the pure tones used to determine the frequency tuning curves also constituted the acoustic CS+ and CS− depending on whether they were preceded by a flashing light (for the CS+) or not. Animals were trained until they reached 90% of responses (bradycardia) to the CS+ and less than 40% of response to the CS−. During off-line tests, single unit recordings were performed in primary auditory cortex at presentation of sequences of CS−/CS+/CS− trials in normal conditions (control), then in the presence of continuous iontophoretic ejections of neuromodulators antagonists. **(A)** Blockage of facilitation by the muscarinic antagonist Atropine. A1. For this cell, the responses obtained in the neuron's frequency receptive field were largely facilitated from 14 to 20 kHz during the CS+ trials (i.e., when the flashing light was presented before the pure tones). When the same sequence of CS−/CS+/CS− was presented in the presence of atropine, these facilitations were no longer observed, indicating that, for this particular cell, the task-related changes were mostly mediated by cholinergic receptors. **(B)** Blockage of facilitation by the noradrenergic antagonist Propranolol. A1. For this cell, the responses obtained in the neuron's frequency receptive field were largely facilitated from 0.2 to 1.1 kHz during the CS+ trials (i.e., when the flashing light was presented before the pure tones). When the same sequence of CS−/CS+/CS− was presented in the presence of Propranolol, the facilitation was no longer observed, indicating that, for this particular cell, the task-related changes were mostly mediated by noradrenergic receptors. **(C)** Group data: on average, task-related changes in evoked responses were blocked by Atropine in 45% of the cells tested (*n* = 52 cases of successful blockages) and by Propranolol in 25% of cells tested (*n* = 45 cases of successful blockages). Unpublished data from Manunta and Edeline.

For years, ACh and NE have been studied using iontophoretic applications and it seems that despite conflicting results these two neuromodulators are able to promote a sharpening of functional properties in sensory cortices. Results obtained with stimulation of source nuclei of these two neuromodulators only partially confirm their opposing effects on evoked responses and tends to confirm that both can favor increased responses. Stimulation of these source nuclei was considered as a mechanism allowing Hebbian rules to operate in sensory cortices (Ahissar et al., 1996; but see Cruikshank and Weinberger, 2001 for discussion). This line of research has promoted a widespread interest in receptive field and map reorganization, triggered by activation of neuromodulatory systems. An important point that deserves consideration is that there is no natural situation during which only a single neuromodulator is involved. In most of the attentional tasks or learning situations, it is quite difficult to dissociate the contribution of the cholinergic, dopaminergic, and noradrenergic system. In such experiments, it is quite difficult to block task-related changes in neuronal activity by application of selective antagonists. In the few cases where such a challenge has been tackled, it was claimed that application of antagonists of a particular neuromodulator prevented task-related changes in neuronal activity (see for example Williams and Goldman-Rakic, 1995; Sawaguchi, 1998, 2001). However, to the best of our knowledge, the effects of antagonists of different neuromodulators were not tested in these experiments. In the guinea pig ACx, we found that, in fact, task-related receptive field changes could be blocked by iontophoretic applications of either cholinergic or noradrenergic antagonists (Figure 5; Manunta and Edeline, unpublished data). Thus, it seems that in a given experimental situation, several neuromodulator antagonists can prevent task-related neuronal activities, suggesting that they act in concert to modulate neuronal responses in behaving animals.

Given the ubiquity of NE and ACh in the entire central nervous system, it can be envisioned that one of the main roles of these neuromodulators is to promote tighter links between sensory processing/perception and higher brain functions such as working memory and executive functions in general. So far, only computational models have pointed out that NE and ACh can have distinct roles in decision-making and/or in the processing of uncertainty (Yu and Dayan, 2002, 2005). Clearly, multi-site electrophysiological recordings in behaving animals can help understand how the links between sensory processing and higher order processing are modulated by NE or ACh. Several techniques such as the directed coherence (DCOH) of local field potential signals provide valuable tools to study directional network interactions. For example, it has been used to reveal that interactions between olfactory bulb and hippocampus are mediated via beta rhythm rather than theta rhythm (Gourévitch et al., 2010).

Whatever refinements will be proposed for future studies to evaluate the interactions between neuromodulators, it is now time to consider that the vast amount of findings that have been documented so far, rely on two assumptions: (i) first that the firing rate is the code that operates in any sensory system and (ii) second that map reorganization is the mechanism by which cognitive processes influence sensory cortex processing. Over the last 10 years, these assumptions have been challenged by compelling evidence indicating that the neural code underlying the perception of complex sounds mostly relies on the temporal organization of neuronal responses rather than on the global firing rate (i.e., the number of action potentials emitted during the stimulus). Studying the modulation induced on the temporal organization of spike trains and the between cell interactions by NE and ACh is still in its infancy and vast avenues of research remained unexplored to address this question. In addition, optogenetic techniques that are now available could be used to selectively activate a particular neuromodulatory system without activating neighboring cells. With this technique, activating cholinergic or noradrenergic terminals within a given cortical area should help clarifying the impact of these two neuromodulators on the neural code. Finally, looking at the effects of neuromodulators on an ensemble of neuronal activity, monitored with optic methods (Ca-dyes or V-sensitive dyes) could be considered although to date these techniques have only been used *in vitro* (e.g., Watanabe et al., 2009).

As in any field, new insights will come with the use of a combination of techniques (optogenetic, large scale ensemble recordings etc…). But it is only if these techniques address functional questions in behaving animals that relevant and long-lasting responses will be achieved.

### Conflict of interest statement

The author declares that the research was conducted in the absence of any commercial or financial relationships that could be construed as a potential conflict of interest.

## Footnotes

^1^In slices of somatosensory cortex, Devilbiss and Waterhouse (2000) have reported that some cells displayed biphasic effects on responses evoked by pulses of glutamate, but no synaptic inputs were tested in this study.

^2^An increased selectivity was also observed in visual thalamus: ACh enhanced the contrast between the responses obtained when a stimulus fell in excitatory vs. inhibitory zones of the receptive field (Sillito et al., 1983).

^3^In this experiment, application of Idazoxan which should increase the extracellular NE concentration (see Edeline, 1995) did not modify the evoked firing rate but increased the between cell cross-correlations.
